# The Hospital Saturday Fund and Street Collections

**Published:** 1896-11-21

**Authors:** 


### THE HOSPITAL SATURDAY FUND AND STREET COLLECTIONS.

THE CHAIRMAN PROPOSES THEIR ABOLITION.

On Saturday evening, 14th inst., a special meeting of the board of delegates of the London Hospital Saturday Fund was held at the Printers' Hall, Holborn Circus, E.C., to consider a proposal by the Chairman of the Fund (Mr. R. B. D. Acland) that the ladies' street collections be discontinued. The meeting was well attended, and the proceedings, which lasted for about three hours, at first threatened to be very stormy. The chairman and some of the earlier speakers, whether for or against the motion, were constantly interrupted, and it was not until the chairman had named the chief disturbers of the meeting, and a proposal that they be "requested to retire" had been made, that the meeting settled down to a quiet discussion of the question. From the very commencement, in spite of an excellent speech from the chairman, it was evident which way the feelings of the majority of those present inclined, and, in the result, an amendment in favour of a continuance of the street collections was declared carried by a large majority.

Before the chairman proposed his resolution,

The Secretary read letters from the Islington, the St. George's, and the Lewisham Local Committees protesting against the resolution.

The Chairman proposed the following resolution:—

"That the ladies' street collection be abolished."

He said that the present was one of the most important occasions in the history of the Fund on which they had ever met, because his resolution involved a change in the work which had been done by the Fund. Before giving the reasons which had induced him to move this resolution, he explained that his motion had taken the form it had because, although it would have been possible to put forward a colourless resolution more gratifying possibly to the feelings of a portion of his audience, yet the matter was one of so much importance that, after due consideration, he thought it was best to go straight to the middle of the question, and to place such a resolution before the meeting as would oblige them to decide whether they intended to continue the system or not. Coming to the question of the street collection, he first of all conceded that all the arguments of a sentimental character were in favour of its continuance, because everyone of any imagination must feel that there was something beautiful in the idea of the gentler and more sympathetic sex going out into the streets once a year and collecting alms on behalf of the sick and suffering. From that point of view the street collection was unimpeachable. But, unfortunately, when they came to look at the matter from the point of view of fact, the stern logic of facts was at the present time against its continuance. Some two years ago he called the attention of the Fund to many reasons why, in his opinion, even then it was desirable that the Fund should face the question, and he expressed his own personal opinion that that form of collecting money for the hospitals ought to be abandoned. Since that time he had seen absolutely nothing to change the opinions he then expressed, viz., that from the point of view of advertisement the street collection had actually done the Fund harm, and from every other point of view it was disadvantageous to the real work of the Hospital Saturday Fund—the collecting for the hospitals of the alms of the working classes in every place of business. More than that, since two years ago the state of affairs had altered very materially. Two facts could not be denied—first, that there had been an enormous increase in the number of street collections Saturday after Saturday; and, secondly, that coupled with the increase of street collection there had been a corresponding and more than a corresponding decrease in the order and dignity and decorum in the way in which these street collections had been conducted. Originally ladies went out into the streets and collected alms once a year, and they were content to let their boxes and their stations make their mute yet eloquent appeal on behalf of the sick and suffering. Now, Saturday after Saturday, this system went on; ladies—or persons who called themselves ladies he supposed—went into the streets, invaded the railway stations, in fact, every place where people congregated, rattled their boxes in the faces of the passers-by, and in some of the more discreditable instances even caught hold of the passers-by in their endeavours to attract attention and to get money. He did not desire to say very much about the effect of this system of street collection upon those who were engaged in it, but his own strong opinion on the subject was that, as things now were, nothing would induce him, on any consideration, to allow any of those near and dear to him to be engaged on this work of street collection. One great result of this flooding of the streets with lady collectors had been to raise in the popular mind a general dislike to this system of street collection. His reasons for saying this were—first, that there was hardly a newspaper of good standing and repute published in London which had not during the past year attacked the system; secondly, the chairman of the County Council, in his annual address to the Council, had deemed it his duty to call attention to this system, and to express his opinion that the gigantic proportions it had now attained must in time lead to prevention by the police; thirdly, questions had been asked in Parliament on the subject, and they were brought face to face with the practical condemnation of the system of the Home Secretary. This condemnation was certainly coupled with the statement that the powers confided to him by law did not prevent its being done, but he expressed a condemnation of a system which, originally admirable and good, had become in its present development unworthy of continuance by such an important body as the Hospital Saturday Fund, for the Fund could not say that the criticisms made applied to everybody else's street collection but its own. (Hear, hear.) In face of this growing dislike, therefore, expressed in the press, in the County Council, and in Parliament, he (the speaker) asked his hearers whether it would be wise and statesmanlike, however great might be their desire to do so, to continue the system of street collection. (Cries of "Yes.") Then the gentlemen who said "Yes" had the most wonderful ideas of statesmanship. The Fund, by its street collections, appealed to the public for voluntary subscriptions, and voluntary contributions could only be obtained to the extent to which the public were willing to give. If, therefore, there was an objection on the part of the public to the form of appeal which was used, but one result could happen, and that was that the street collection would go by the board. The street collection was now £1,000 lower than it was four years ago. During that four years it had, with the exception of the present year, shown a steady decrease, and if the public continued to object it would go down steadily until it vanished. In his opinion, therefore, it would be more statesmanlike and wiser to say that: "Seeing that, what was an excellent form of collection, has become abused and brought to bad repute by others, we who initiated it will be the first to try and purge the streets of what is now looked upon as a nuisance." Taking the home points of view, Mr. Acland said that there was abundant evidence that the position of the lady collectors, in some districts at all events, had become an unpleasant one. First, because of the remarks that were made at them and to them; and, secondly, because the assistance which was, in the first instance, willingly given was now grudgingly given by tradesmen and others. (Hear, hear, and cries of "No.") These two reasons had rendered it more difficult each year to find suitable lady collectors. (Hear, hear.) Of course, the ladies who had begun at the beginning and had continued their services throughout were now as good as they were at the beginning, but unfortunately their ranks had become thinned from various causes, and it was found increasingly difficult to find lady collectors of suitable standing and position to do the work—(Voice: "That is true!")—and consequently each year it was found that the quality of the ladies who were willing to offer themselves deteriorates. ("No, no.") The very amendment which was to be moved that night supported that view, as it assumed that the street collection had lowered in tone, for it commenced, "It is very desirable that the tone of our street collection should be raised in every direction possible." Another objection to the street collection was that it was one of the most extravagant ways of getting money. ("No, no.") An exact account had been kept for the last year of the expenses of the street collection, and after allowing a sum of £77 7s. 6d. for wages of the staff—a most moderate allowance—the total actual expenses of the street collections were £852 3s. 2d. Most of them had deluded themselves into the idea that the account which appeared in the balance sheet of local committee expenses was the cost of the street collection, but it was nothing of the sort. The total amount collected was about £4,780; so that the expenses of the street collection came to about 18 per cent. of the amount collected. A society like their own, which wanted to keep hospital expenditure within reasonable limit, could not defend for a moment an expenditure of that sort. What would their Distribution Committee say to any hospital which collected its money at an expense of 18 per cent.? Besides this, the enormous expense of the street collection made the cost of working the Fund over 2 per cent. of the amount collected more than it would otherwise have been. That was to say, it raised their expenses from under 10 per cent. of the amount collected (the amount at which the workshop collection was worked) to 12 per cent. (the amount which both the workshop and the street collections came out at). This form of collection was also extremely expensive, because it distracted the attention of the staff from what, in his opinion, was the real work of the Hospital Saturday Fund—the organising of the workshop collections. The organising of the street collections took about three months in the year; that was to say, for three months of the most valuable part of the year the staff was taken away from what was the permanently useful work of getting the shops to contribute. The street collection also was an absolute hindrance to the Fund in its work among the workshops, as many shops declined to contribute on account of it. This point of view Mr. Acland supported by reading a letter on the point from the organising secretary, Mr. A. W. Davis, in which instances were given of permission to organise a collection among workpeople being refused because of the street collection. Finally, what did the Fund actually realise from the street collection? Could they afford to give it up? First of all, it must be remembered that in return for their share in getting up the street collection the local committees were entitled to letters of recommendation for hospitals. The total street collection this year was £4,780, and deducting from that £850 for expenses the net result was £3,930. Last year—for, unfortunately, the figures for this year were not available—2,333 letters of recommendation were issued to the local committees in return for their contributions, or one letter for every 32s. contributed. Each letter cost the Fund just about £1, so that the actual gain to the Fund from the street collections was about 12s. on every letter, or in round figures £1,300. So that the Fund really received about £1,300 for the street collection, as against the £16,000 to £17,000 it got from the workshops. Was it worth while to run counter to the opinion of a very large and influential section of the public for the sake of some £1,300 a year? It was asked what could be done to raise money for the Fund in place of the street collection? He had shown that there was only a sum of £1,300 to be made up, and he was sure that if the organising staff gave up the time now devoted to the street collection to further work among the workshops that sum would easily be made up. Besides which their putting a stop to these collections would enormously improve the position of the Fund among the thoughtful philanthropic and charitable people in London. The Fund would have been the first great organisation to recognise the fact that the time for street collections had passed, just as it in its wisdom had been the first to see that collecting in the streets was a good thing. In conclusion, Mr. Acland appealed to his hearers to well consider whether the Fund should not voluntarily give up street collecting before they were forced to do so.

Mr. H. N. Hamilton-Hoare (treasurer), in seconding the motion, said that he looked at this matter in a very commonplace way. What the chairman proposed was a reform. Now all his hearers knew that when a reform was suggested there was a very large number of people who at once lifted up their hands, and said, "Are you going to suggest a thing like that? It will upset everything and everybody." The next year those same people came to the reformer and said, "I think there is something in what you said last year." The following year they agreed to it. (Laughter.) So with this proposal. He did not expect it would be carried that evening—(applause)—but next year he expected their opponents would say there was something in it, and the next year they would propose and pass it. (Laughter and applause.)

The discussion which followed lasted for about two hours, the first speaker alone taking up, in spite of several attempts to howl him down, about three-quarters of an hour, after which it was moved and carried, "That future speakers be limited to five minutes." The speakers were, with one exception, opposed to the motion, the principal points made being as follows: (1) That the street collection was an excellent advertisement for the Hospital Saturday Fund; (2) that the objection on the part of the public and tradespeople was exaggerated, and came chiefly from those who did not wish to give; (3) that the falling off has been partly caused by bad trade and by the fact that the Fund itself has been divided on the subject; (4) that the discontinuance of the street collection would lead to the abolition of the local committees, who had done so much in the past to promote the workshop collections; (5) that this last point would probably result in the formation of numerous small Saturday Fund organisations in London in place of one large one; (6) that the street collection afforded an opportunity for giving to people who would not otherwise give; (7) that although the fund itself might only get £1,300 out of the street collection, yet the hospitals benefited by the balance, and the collecting of money for the hospitals was one part of the work of the Fund; and (8) that the time was not yet ripe for giving up the street collection.

In the end the following amendment, proposed by Mr. W. H. Baker, was carried by a large majority:—

"It is very desirable that the tone of our street collection should be raised in every direction; and the Council is directed to draw up rules at once with this object. That the secretaries of local committees be invited to attend the special meetings of the Council called for this purpose, and be invested with voting power."

